# Multi-omics analysis elucidates the therapeutic mechanisms of the Quzhi formula in metabolic dysfunction-associated steatohepatitis targeting gut microbiota, lipid metabolism, and the role of its metabolite fraxin

**DOI:** 10.3389/fphar.2025.1694242

**Published:** 2025-11-03

**Authors:** Jiao-Xiang Wu, Yue-Lan Wu, Mei-Fang Li, Nian Liu, Ying Liu, Yan-Ping Huang, Yuan Gan, Xiao-Yu Wang, Hai-Sheng Chai, Jin Xu, Qian Xi, Xi-Rong Guo, Hui-Ming Sheng, Ting-Ting Shen, Qin Zhang

**Affiliations:** ^1^ Phase I Clinical Trial Unit and Department of Infectious Diseases and Department of Clinical Laboratory and Plastic Surgery Department, Tongren Hospital, Shanghai Jiao Tong University School of Medicine, Shanghai, China; ^2^ Henan University, Zhengzhou, China; ^3^ eHealth Program of Shanghai Anti-doping Laboratory, Shanghai University of Sport, Shanghai, China; ^4^ Clinical College of Traditional Chinese Medicine, Gansu University of Chinese Medicine, Lanzhou, China; ^5^ Yueyang Integrated Chinese and Western Medicine Hospital, Shanghai University of Traditional Chinese Medicine, Shanghai, China; ^6^ Department of Endocrinology, Shanghai Pudong New Area Gongli Hospital, Shanghai, China

**Keywords:** metabolic dysfunction-associated steatohepatitis (MASH), Quzhi formula (QZF), fraxin, AMPK, autophagy

## Abstract

Metabolic dysfunction-associated steatohepatitis (MASH) is an advanced stage of fatty liver disease with no approved pharmacotherapies. The Quzhi Formula (QZF), a traditional Chinese medicine utilized clinically for nearly two decades, has shown promising efficacy against MASH; however, its mechanisms of action remain largely unexplored. To elucidate these mechanisms, we conducted a multi-omics investigation integrating 16S rRNA sequencing, untargeted metabolomics, and transcriptomics in a MASH mouse model, with findings validated by histology. QZF treatment significantly alleviated hepatic steatosis, restored gut microbial diversity, and suppressed the proliferation of *Enterococcus*, a genus implicated in MASH pathogenesis. Transcriptomic and metabolomic analyses demonstrated that QZF’s therapeutic effects were mediated through the regulation of lipid metabolic pathways and the activation of autophagy. Furthermore, we identified fraxin as a pivotal bioactive metabolite contributing to QZF-induced autophagy. Our study demonstrates that QZF ameliorates MASH in a concerted manner by remodeling the gut microbiota, reprogramming hepatic metabolism, and promoting autophagy via fraxin. These results provide a comprehensive mechanistic foundation for QZF as a multi-targeted therapeutic candidate for MASH.

## 1 Introduction

Metabolic dysfunction-associated fatty liver disease (MAFLD), formerly known as non-alcoholic fatty liver disease (NAFLD), represents the predominant form of chronic liver disease worldwide, with its estimated prevalence having risen to 38.2% (Wong et al., 2023). Metabolic dysfunction-associated steatohepatitis (MASH), a distinct pathological subtype of MAFLD (Rao et al., 2023; Tamimi et al., 2024), is characterized as an inflammatory condition featuring simple hepatocellular steatosis, portal or central vein inflammatory cell infiltration, and hepatocyte ballooning (Hags et al., 2024; Wu et al., 2021; Qu et al., 2019). As the advanced stage of the MAFLD disease spectrum, MASH progresses to hepatic fibrosis in 40.76% of cases and drives a marked increase in cirrhosis prevalence (Miao et al., 2024). It is projected to surpass viral hepatitis as the primary indication for liver transplantation and the leading etiology of hepatocellular carcinoma in the future (Younossi et al., 2025). Furthermore, MASH-associated metabolic dysregulation correlates with elevated risks of severe late-stage hepatic complications (Jarvis et al., 2020). Currently, only one FDA-approved therapeutic agent for MASH is available (Kokkorakis et al., 2024), leaving the substantial medical need for preventing, halting, or reversing MASH largely unmet.

Emerging evidence underscores MAFLD as a multifactorial disorder arising from systemic metabolic dysregulation across organ systems. Central to its pathogenesis are interconnected risk factors including hepatic nutrient overload (particularly carbohydrates/lipids), accumulation of lipotoxic metabolites, oxidative stress, immune activation, endoplasmic reticulum stress, and genetic predisposition (Buzzetti et al., 2016; Younossi et al., 2018). This complexity manifests through a “*multiple-hit*” pathological framework characterized by concurrent hepatocellular lipid accumulation, insulin resistance, oxidative DNA damage, inflammatory cascades, and fibrotic remodeling (Kuchay et al., 2020). Gut microbiota dysbiosis is intricately linked to the pathogenesis of MASH. Under the synergistic influence of environmental risk factors and genetic predisposition, crosstalk among the liver, adipose tissue, and gut microbiota drives systemic metabolic dysfunction and insulin resistance, resulting in enhanced hepatic fatty acid influx and *de novo* lipogenesis (Leung et al., 2016). Furthermore, autophagy—a conserved cellular degradation pathway enabling the recycling of proteins and organelles—has emerged as a critical regulator in MASH progression (Ren et al., 2024).

Recent studies have shown that traditional Chinese medicine can be used as a potential drug for the treatment of MASH (Liang et al., 2025; Xu et al., 2024; Gong et al., 2022; Tang et al., 2024), such as the *Xiaoji-chenpi* formula (XCF) can improve lipid metabolism, inhibite inflammation, and reduce liver fibrosis in MAFLD (Liang et al., 2025). *Gan-jiang-ling-zhu* decoction improves steatohepatitis by regulating gut microbiota-mediated 12-tridecenoic acid inhibition (Xu et al., 2024). The Quzhi Formula (QZF), a traditional Chinese medicine, emerges here as a further potential therapeutic for metabolic dysfunction-associated steatohepatitis (MASH). This clinically optimized formulation has been systematically employed in our therapeutic practice for nearly two decades, demonstrating consistent and significant clinical efficacy in disease management (Xie et al., 2010; Chai et al., 2020; Wu et al., 2022). The QZF is composed of three medicinal botanicals: *Polygonum cuspidatum* Siebold and Zucc. (Hu zhang), *Senna obtusifolia* (L.) H. S. Irwin and Barneby (Jue mingzi), and *Crataegus pinnatifida* Bunge (Shan zha) (Wu et al., 2022). *Polygonum cuspidatum* Siebold and Zucc, serving as the sovereign botanical drug in this formulation, contains multiple bioactive metabolites—including *quinones*, *stilbenes*, and *flavonoids*—that exert multifaceted therapeutic effects on hyperlipidemia, inflammatory cascades, microbial infections, and oncogenesis through distinct pharmacological mechanisms (Peng et al., 2013). *Senna obtusifolia* (L.) H. S. Irwin and Barneby, another critical botanical drug, demonstrates broad-spectrum bioactivities encompassing antidiabetic, anti-inflammatory, antineoplastic, antimutagenic, and hepatoprotective properties (Yadav et al., 2010). Meanwhile, *C. pinnatifida* Bunge is extensively documented in both preclinical and clinical contexts for its efficacy in modulating metabolic syndrome-related pathways (Dehghani et al., 2019).

AMP-activated protein kinase (AMPK) serves as a central regulator of cellular energy metabolism and represents a critical therapeutic target for MASH intervention (Wang et al., 2025). And many natural bioactive metabolites demonstrate promising potential for the treatment of MASH by regulating AMPK pathway. In this study, we demonstrate that both QZF and Fraxin also activate AMPK and ameliorate lipid accumulation *in vitro*.

Collectively, QZF is rationally designed based on the pathophysiological characteristics of MASH, integrating optimized phytochemical synergies to target multifactorial disease mechanisms. In this study, integrated multi-omics analyses reveal that QZF alleviates MASH by modulating the gut-liver axis to restore microbial diversity, suppress pro-steatotic *Enterococcus*, and activate autophagy-driven metabolic reprogramming, thereby reducing hepatic lipid accumulation and positioning QZF as a systems-level therapeutic for metabolic disorders.

## 2 Materials and methods

### 2.1 Botanical drugs materials and extracts preparation

#### 2.1.1 Botanical drugs and authentication

The botanical drugs in QZF are detailed in Table 1, which includes their scientific and species names, pharmaceutical names, Chinese phonetic names, and origins. The formula consists of three botanical drugs: *P. cuspidatum* Siebold and Zucc. (20 g), *S. obtusifolia* (L.). H. S. Irwin and Barneby (30 g), and *C. pinnatifida* Bunge (30 g). These botanical drugs were sourced from the Shanghai Wanshicheng Pharmaceutical Co., Ltd., (Shanghai, China) or Shanghai Kangqiao Chinese MedicineTablet Co., Ltd., (Shanghai, China) and authenticated by professor Wei Liu, Department of Pharmacy, Shanghai University of Traditional Chinese Medicine.

**TABLE 1:** The botanical drug(s) of QZF.

Scientific name	Pharmaceutical name	Latin name	Chinese pin yin	Place of origin (commercial supplier/province)
*Senna obtusifolia* (L.) H. S. Irwin and Barneby	Sickle Senna Seed	Cassiae Semen	Jue mingzi	Shanghai Wanshicheng Pharmaceutical Co., Ltd. (Shanghai, China)
*Polygonum cuspidatum* Siebold and Zucc.	Giant Knotweed Root	Polygoni Cuspidati Rhizoma et Radix	Hu zhang	Shanghai Kangqiao Chinese MedicineTablet Co., Ltd. (Shanghai, China)
*Crataegus pinnatifida* Bunge	Chinese Hawthorn Fruit	Crataegi Fructus	Shan zha	Shanghai Kangqiao Chinese MedicineTablet Co., Ltd. (Shanghai, China)

The voucher specimen of *P. cuspidatum* Siebold and Zucc (Huzhang) is deposited in the Herbarium of the College of Pharmacy, Guizhou University of Traditional Chinese Medicine (Guizhou, China), with the voucher number GZTM0094265. It was collected and preserved by Hou Xiaoqi on 12 June 2019, in Guizhou, China. The specimen of *S. obtusifolia* (L.) H. S. Irwin and Barneby (Juemingzi) is housed in the Plant Herbarium of the School of Geography and Environmental Science, Guizhou Normal University (Guizhou, China), under the voucher number GNUG0008259. It was collected and preserved by Wang Yuanhong on 22 November 2017, in Guizhou, China. The specimen of *C. pinnatifida* Bunge (Shanzha) is stored in the Medicinal Plant Herbarium of the College of Pharmacy, Jiamusi University (Heilongjiang, China), with the voucher number JMSMC0005742. It was collected and preserved by Wang Lihong on 17 June 2023, in Heilongjiang, China.

#### 2.1.2 Botanical drugs extract preparation

According to the specified ratio, 260 g of *P. cuspidatum* Siebold and Zucc., 390 g of *S. obtusifolia* (L.) H. S. Irwin and Barneby and 390 g of *C. pinnatifida* Bunge were taken. For the first extraction, 10 times the amount of water was added, and reflux extraction was performed for 2 h. For the second extraction, 8 times the amount of water was added, and reflux extraction was conducted for 1 h. The extract was filtered through a 200-mesh sieve, and the filtrate was concentrated to a relative density of 1.07, yielding approximately 1.825 kg. Under gentle stirring, 3.118 kg of ethanol was added to achieve an alcohol concentration of 60%. The mixture was then purified by standing for over 12 h. The supernatant was collected and concentrated to approximately 500 mL (density 1.154). This concentration was used for subsequent mass spectrometry analysis and animal administration.

#### 2.1.3 Chemical profiling and quality control of QZF

To identify the metabolites of QZF extract, we performed LC-MS/MS, the results were presented in Supplementary Table S1. The LC–MS fingerprint of the QZF extract was established by identifying characteristic peaks in both positive and negative ion modes as described by Fan et al. (2022). The peak areas were recorded, and the relative standard deviations (RSD) for the relative retention time and relative peak area were calculated as 0.00%–0.22% and 1.06%–1.43%, respectively, indicating good instrument precision. The cosine similarity algorithm was applied to calculate the similarity between all samples and the common peaks established based on all samples. Detailed fingerprint similarity results among samples can be found in the inter-sample similarity analysis table. Meanwhile, the cosine similarity algorithm was also used to compute the similarity between all samples and the common peaks established within different sample groups. These corresponding results are provided in Supplementary Figure S1 and the ConPhyMP-checklist were presented in Supplementary Table S2.

### 2.2 MASH model induction and QZF administration

As we described in 2022 (Wu et al., 2022), six-week-old male C57BL/6J mice (n = 45) were used for all experiments. MASH was induced by feeding mice a choline-deficient, l-amino acid–defined, high-fat diet (CDAHFD, Dyets, CD-HF60)^11,12^ for 3 weeks. Mice were randomly assigned to five groups: control (normal chow, n = 9), model (MASH, n = 9), QZF8.3331 g/kg -treated (MASH + QZF, n = 9), QZF4.1666 g/kg -treated (MASH + QZF, n = 9), and QZF-toxicity (normal chow + QZF 8.3331 g/kg, n = 9).

QZF was administered orally at a dose of 8.3331 g/kg/day or 4.1666 g/kg/day for 4 weeks following the establishment of the MASH model. The composition and dosage were based on previous clinical and pharmacological studies.

The mice were then sacrificed, and intestinal contents and liver samples were collected for analysis. Anesthesia was induced via intraperitoneal injection of 0.3% pentobarbital sodium at a dosage of 0.05 g/kg. Then the blood and liver samples were collected. All animal experiments were conducted in accordance with institutional ethical guidelines.

### 2.3 Liver tissue histological and lipid staining

Hepatic tissue specimens underwent immersion-fixation in 4% paraformaldehyde (24 h), followed by standard paraffin embedding procedures. Thin sections (5 μm) were prepared and subjected to hematoxylin and eosin (H&E) staining protocol to enable microscopic morphological analysis. Frozen liver sections prepared at −20 °C cryostat temperature underwent Oil Red O staining (0.3% working solution, 37 °C incubation) for specific detection of lipid deposits. Images were captured using a brightfield microscope.

### 2.4 Gut microbiota analysis by 16S rRNA sequencing

Fecal samples were collected and subjected to microbial DNA extraction using the HiPure Soil DNA Kit or HiPure Stool DNA Kit (Magen, Guangzhou, China) in accordance with the manufacturer’s protocols. The 16S rDNA hypervariable regions of the ribosomal RNA gene were amplified via polymerase chain reaction (PCR). The primers used were listed in Table 2. Each 50 μL PCR mixture contained 10 μL of 5× Q5^®^ Reaction Buffer, 10 μL of 5× Q5^®^ High GC Enhancer, 1.5 μL of 2.5 mM dNTPs, 1.5 μL of each primer (10 μM), 0.2 μL of Q5^®^ High-Fidelity DNA Polymerase, and 50 ng of template DNA. All PCR reagents were obtained from New England Biolabs (USA).

**TABLE 2:** 16S sequence related primer information.

Type	Region	Primer name	Primer sequence	Product length
16S	V4	515F	GTGYCAGCMGCCGCGGTAA	∼292
		806R	GGACTACNVGGGTWTCTAAT	
16S	V3-V4	341F	CCTACGGGNGGCWGCAG	∼466
		806R	GGACTACHVGGGTATCTAAT	
16S	V4-V5	515F	GTGCCAGCMGCCGCGGTAA	∼412
		907R	CCG​TCA​ATT​CCT​TTG​AGT​TT	
16S	V5-V7	799F	AACMGGATTAGATACCCKG	∼414
		1193R	ACGTCATCCCCACCTTCC	
16S	V4-V5	Arch519F	CAGCMGCCGCGGTAA	∼416
		Arch915R	GTG​CTC​CCC​CGC​CAA​TTC​CT	
18S	V4	528F	GCG​GTA​ATT​CCA​GCT​CCA​A	∼260
		706R	AATCCRAGAATTTCACCTCT	

The resulting amplicons were excised from 2% agarose gels and purified with the AxyPrep DNA Gel Extraction Kit (Axygen Biosciences, Union City, CA, USA) following the manufacturer’s instructions. The purified products were quantified using the ABI StepOnePlus Real-Time PCR System (Life Technologies, Foster City, USA). Equimolar amounts of the amplicons were pooled and subjected to paired-end sequencing (PE250) on an Illumina platform according to standard protocols. Sequencing data were subsequently analyzed with QIIME2 and LEfSe to assess microbial diversity, relative abundance, and identify potential biomarkers.

### 2.5 Untargeted metabolomics analysis

Liver tissues were harvested, homogenized, and extracted using methanol/acetonitrile mixtures. Metabolomic profiling was performed using UHPLC-Q-TOF-MS under both positive and negative ion modes. Analysis was performed using an UHPLC (1290 Infinity LC, Agilent Technologies) coupled to a quadrupole time-of-flight (AB Sciex TripleTOF 6600) in Shanghai Applied Protein Technology Co., Ltd. For HILIC separation, samples were analyzed using a 2.1 mm × 100 mm ACQUIY UPLC BEH 1.7 µm column (waters, Ireland). For RPLC separation, a 2.1 mm × 100 mm ACQUIY UPLC HSS T3 1.8 µm column (Waters, Ireland) was used. Data were processed with XCMS and annotated against the HMDB and KEGG databases. Multivariate statistical analyses (PCA, OPLS-DA) were conducted to identify differential metabolites and enriched pathways.

### 2.6 RNAseq of liver tissue

Total liver RNA was isolated from hepatic tissues using Trizol reagent. RNA quantification was precisely determined using Qubit 4.0 Fluorometer. After rigorous quality control, strand-specific transcriptome libraries were prepared following Illumina TruSeq Stranded mRNA protocol and sequenced on Illumina Novaseq 6000 platform with paired-end 150 bp (PE150) configuration, generating raw sequencing data in FASTQ format. Differentially expressed genes were defined by fold change >1.5 and p < 0.05, and subjected to GO and KEGG enrichment analysis.

### 2.7 Cell viability measurement

AML12 cells were incubated in AML12 Cell Specific Medium (Wuhan Pricella Biotechnology, CM-0602) with or without various concentrations of Fraxin (TargetMol, USA, T3783) for 24 h. The cell viability was detected using CCK-8 assay kit (TargetMol, USA, C0005) as the manufacture’s instruction.

### 2.8 Cell Oil Red O staining

To evaluate the intracellular lipid accumulation, Cell Oil Red O Staining was performed. HepG2 cells were first treated with 100 µM Oleic acid (OA) and 50 µM Palmitic acid (PA), followed by 24 h co-exposure to Fraxin at 40 or 60 µM concentrations, with 10 µM resmetirom serving as the positive control. HepG2 cells were fixed in 4% paraformaldehyde for 0.5 h and stained by Oil Red O solution for 0.5 h. Oil red O stained hepatocytes were rinsed with 60% isopropanol and counterstained with hematoxylin. After being washed with distilled water, the stained lipid droplets within cells were observed under an inverted microscope.

### 2.9 BODIPY 493 staining

HepG2 cells were fixed with 4% paraformaldehyde (15 min) and subsequently stained with BODIPY 493 fluorescent dye (20 min incubation). Following three PBS washes to remove unbound probes, intracellular lipid droplets were visualized under a fluorescence microscope, with image acquisition performed using consistent exposure parameters across experimental groups.

### 2.10 Cell lipid measurement

AML12 cells were first exposed to 100 µM OA and 50 μM PA for 24 h, followed by 24-h treatment with Fraxin at graded concentrations (40 and 60 µM) or 10 µM Resmetirom. Subsequently, cellular lysates were collected by centrifugation. Hepatic lipid profiles were quantified using commercially available enzymatic assay kits (Total Cholesterol: A111-2-1; Triglycerides: A110-2-1, Nanjing Jiancheng Bioengineering Institute) according to the manufacturer’s protocols, with optical density measurements normalized to total protein content.

### 2.11 Western blot

Cellular samples were homogenized with ice-cold RIPA lysis buffer (Thermo Scientific, Cat# 89900) containing protease/phosphatase inhibitor to extract the total proteins. Then, the extracted protein samples were separated using 10% SDS-PAGE and transferred onto polyvinylidene fluoride (PVDF, Millipore, IPVH00010) membranes. After blocking with 5% non-fat milk, the membranes were incubated with primary antibodies (Table 3) at 4 °C overnight. Following incubated corresponding secondary antibodies for 2 h at room temperature, the protein bands were visualized using Omni ECL reagent (EpiZyme, SQ101 or SQ201).

**TABLE 3:** Antibody used for western blot.

Protein	Manufacturer	Product number	Source
ASK1	CST	3762S	Rabbit
Phospho-JNK	proteintech	80024-1-RR	Rabbit/IgG
JNK	proteintech	66210-1-Ig	Mouse/IgG1
P62/SQSTM1	proteintech	18420-1-AP	Rabbit/IgG
BAX	proteintech	50599-2-Ig	Rabbit/IgG
Bcl2	proteintech	26593-1-AP	Rabbit/IgG
LC3B	proteintech	18725-1-AP	Rabbit/IgG
ATG7	proteintech	10088-2-AP	Rabbit/IgG
NRF2	CST	12721S	Rabbit/IgG
AMPKα	CST	2532S	Rabbit/IgG
AMPKβ1/2	CST	4150S	Rabbit/IgG
Phospho-AMPKα	CST	2535S	Rabbit/IgG

### 2.12 qPCR

The total RNA was extracted from liver or hepatocytes with Trizol reagent (Sangon Biotech, B511311-0100), following the manufacturer’s instruction. The cDNA synthesis was performed using a reverse transcriptase kit (Takara, RR047). The mRNA levels of genes involved in inflammation and Lipid metabolism were estimated by RT-PCR using an SYBR Green qPCR Master Mix kit (Takara, RR820B). The primer sequences used to amplify mRNA were shown in Table 4.

**TABLE 4:** RT-PCR primer sequences.

Gene	Primer	Sequence
ASK1	Forward	5′-CTG​CAT​TTT​GGG​AAA​CTC​GAC​T-3′
ASK1	Reverse	5′-AAG​GTG​GTA​AAA​CAA​GGA​CGG-3′
P62	Forward	5′-GAC​TAC​GAC​TTG​TGT​AGC​GTC-3′
P62	Reverse	5′-AGT​GTC​CGT​GTT​TCA​CCT​TCC-3′
Actin	Forward	5′-TTG​TTA​CAG​GAA​GTC​CCT​TGC​C-3′
Actin	Reverse	5′-ATG​CTA​TCA​CCT​CCC​CTG​TGT​G-3′
GAPDH	Forward	5′-GTC​AAG​GCT​GAG​AAC​GGG​AA-3′
GAPDH	Reverse	5′-AAA​TGA​GCC​CCA​GCC​TTC​TC-3′

### 2.13 Molecular docking

The three-dimensional structures of core autophagy pathway targets were retrieved from the UniProt database (https://www.uniprot.org/). The three-dimensional structure of Fraxin was obtained from the PubChem database (https://pubchem.ncbi.nlm.nih.gov/). Protein structures were prepared using PyMOL software, involving hydrogen addition, water removal, and ligand separation. Grid box parameters were optimized in AutoDock Tools. Molecular docking simulations were subsequently performed using AutoDock Vina to quantitatively assess the binding modes between the small molecule drug and the core targets. The resulting docking poses were visualized using PyMOL.

### 2.14 Statistical analysis

Data were expressed as mean ± SEM. Comparisons between groups were performed using one-way ANOVA followed by Tukey’s *post hoc* test. Statistical significance was set at *p* < 0.05. All analyses were performed using GraphPad Prism and R software.

## 3 Results

### 3.1 QZF remodeled gut microbiota composition in MASH mice

**FIGURE 1:**
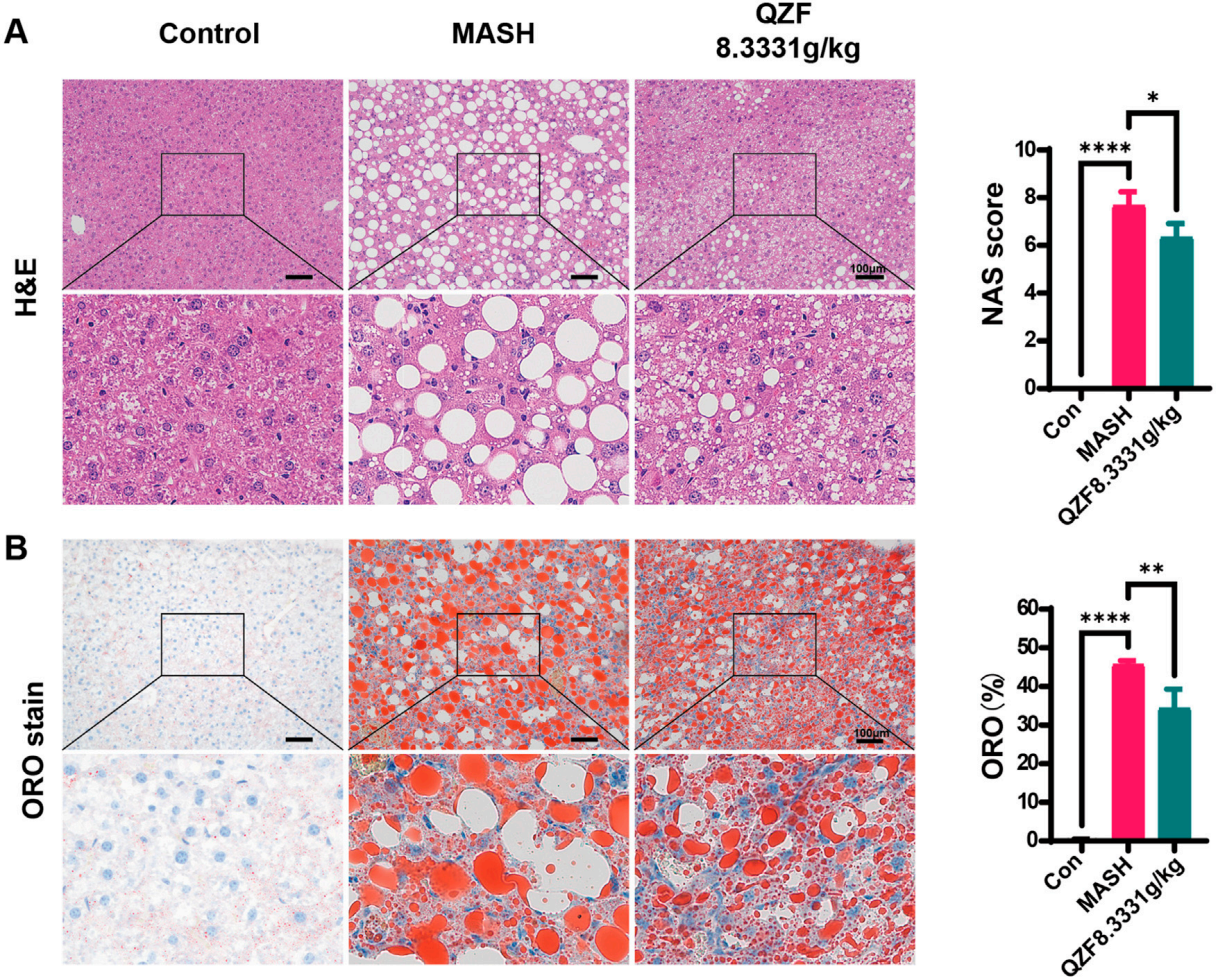
QZF alleviated hepatic steatosis in MASH mice. Male C57BL/6 SPF mice (6 weeks old) were maintained on CDAHFD for 3 weeks and subsequently administered the QZF (8.3331 g/kg) or PBS vehicle by daily gavage for 4 weeks. At the end of week 7, mice were euthanized and liver samples were obtained. Liver sections subjected to **(A)** H&E or **(B)** Oil Red O staining are shown representatively. NAFLD Activity Score (NAS) and representative Oil Red O-stained liver sections with corresponding semi-quantitative analysis of lipid deposition performed using ImageJ. **p < 0.01, ***p < 0.001, ****p < 0.0001vs. model group (one-way ANOVA). Abbreviation: MASH, metabolic associated steatohepatitis; CDAHFD, choline-deficient, l-amino acid–defined, high-fat diet; H&E, hematoxylin and eosin.

The therapeutic potential of the QZF was evaluated in a mouse model of MASH induced by a CDA-HFD diet. Treatment with the high dose of QZF (8.3331 g/kg) significantly alleviated hepatic steatosis and markedly attenuated lipid-associated inflammation compared to the model group (Figures 1A,B). Although the low-dose QZF group (4.1666 g/kg) showed a trend toward improvement, the effect did not reach statistical significance (Supplementary Figure S2). Furthermore, a dedicated acute toxicity study confirmed that the high-dose QZF regimen was well-tolerated, showing no signs of toxicity or histopathological damage to vital organs (Supplementary Figure S3), thereby supporting its safety profile.

**FIGURE 2:**
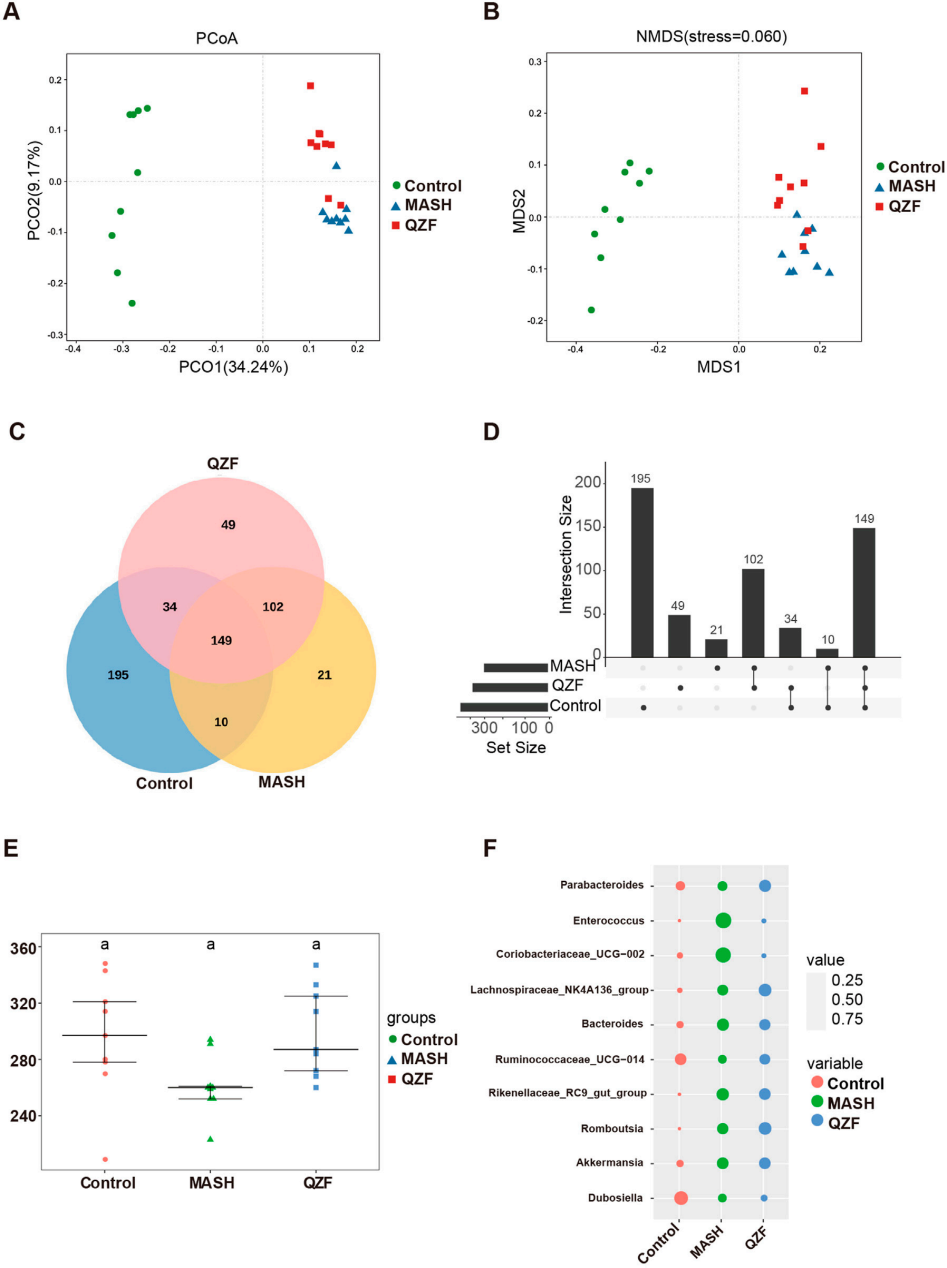
QZF improves microbial diversity and composition in MASH mice. **(A)** Principal coordinate analysis (PCoA) and **(B)** non-metric multidimensional scaling (NMDS) plots, both based on Bray-Curtis dissimilarity, illustrate distinct clustering patterns between the QZF-treated and vehicle-control groups. **(C)** Venn diagram and **(D)** UpSet plot depicting shared and unique bacterial OTUs across Control, MASH, and QZF groups. **(E)** Alpha diversity (observed OTU counts) across Control, MASH, and QZF groups. **(F)** Differential abundance of MAFLD-associated genera revealed by taxonomic profiling.

To investigate the impact of QZF on gut microbial diversity in MASH, we conducted 16S rRNA sequencing of fecal samples. The α-diversity analysis showed a significant increase in microbial richness and evenness following treatment, indicating a restoration of gut microbial homeostasis (Supplementary Figure S4). Principal coordinate analysis further revealed that the microbial community structure in the QZF-treated group was markedly separated from that of the MASH model group (Figures 2A,B). To investigate the shared and unique bacterial taxa among the three experimental groups (Control, MASH, and QZF), we performed OTU-based comparative analyses using both Venn and UpSet plots. A total of 149 OTUs were commonly shared across all three groups, representing a core microbial community. Group Control exhibited the highest number of unique OTUs (n = 195), followed by group QZF (n = 49) and group MASH (n = 21), suggesting substantial group-specific microbial signatures.

In addition to these unique taxa, 34 OTUs were found to be shared exclusively between groups Control and QZF, and 10 OTUs were shared between groups Control and MASH. Notably, 102 OTUs were shared between groups QZF and MASH but were absent in group Control, indicating potential similarities in microbial composition or response patterns between these two groups (Figures 2C,D).

To assess microbial richness and complexity, alpha diversity was evaluated across the three groups using observed OTU counts (Figure 2E). Further analysis of genus-level microbial composition revealed several taxa associated with NAFLD progression (Figure 2F). Notably, the relative abundance of *Enterococcus* was markedly elevated in group MASH compared to groups Control and QZF. In contrast, the abundance of *Enterococcus* was relatively suppressed in group QZF, suggesting a potential protective effect of the intervention applied in this group. These results collectively highlight both the conserved and distinct metabolites of the gut microbiota under different conditions.

The PCoA of the microbiota revealed that, while the microbial community in the QZF treatment group was distinct from that of the MASH group, it remained more similar to the MASH group than to the control. This indicates that the amelioration of MASH by QZF is only partially mediated by remodeling the gut microbiota, suggesting that other mechanisms, such as those involving metabolic regulation, are also likely involved.

### 3.2 QZF modulates hepatic transcriptome and activates AMPK/autophagy-related metabolic pathways

**FIGURE 3:**
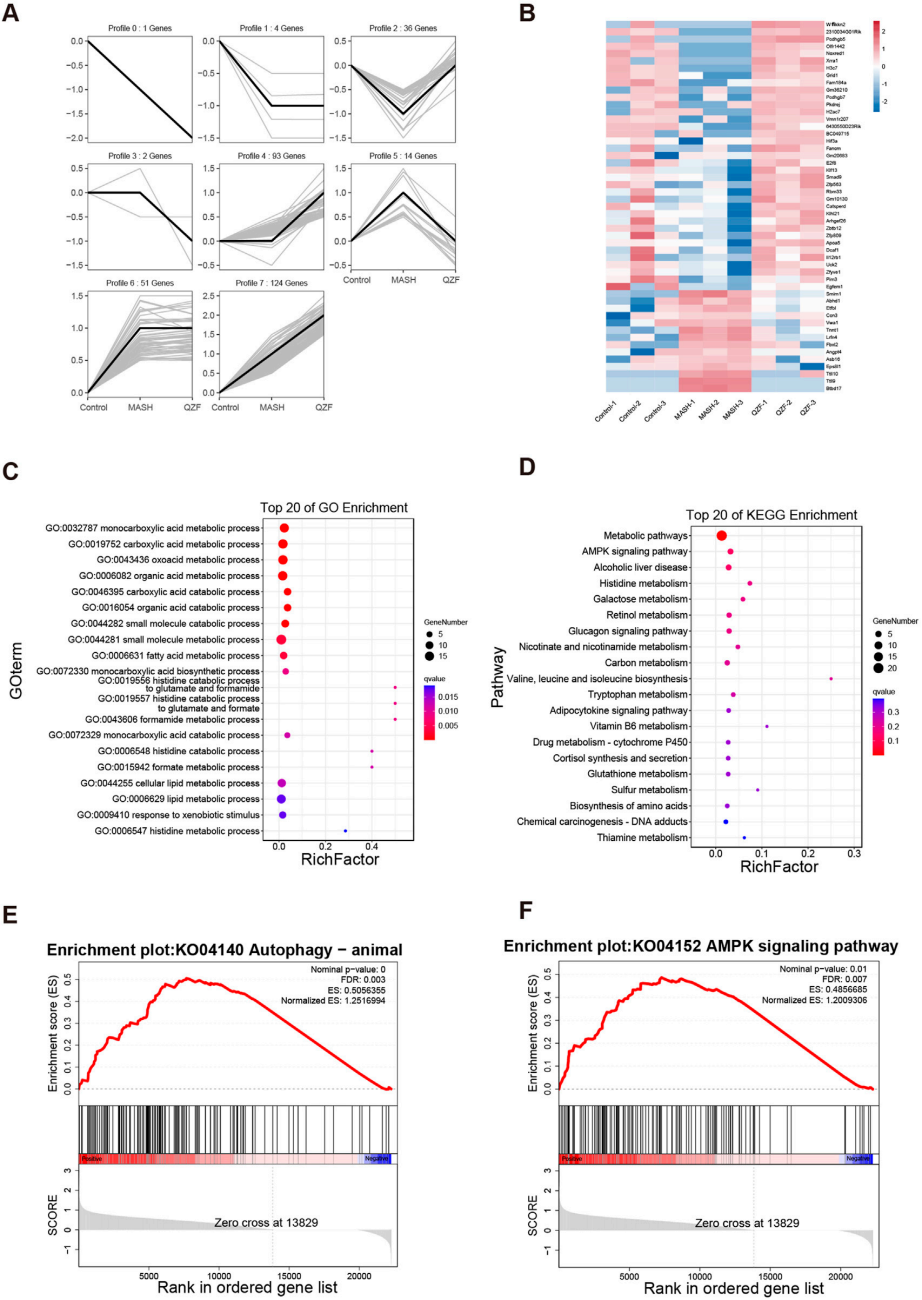
QZF Modulates Hepatic Transcriptome and Activates AMPK/Autophagy-Related Metabolic Pathways. RNA-seq analysis of QZF mechanisms in MASH. **(A)** Gene clustering into eight profiles based on expression trends across Control, MASH and QZF groups. **(B)** Reversion of Profiles 2 and 5 to normal transcriptional states post-QZF treatment. **(C,D)** Metabolic pathway enrichment via GO and KEGG analyses. **(E,F)** GSEA showing enrichment of autophagy and AMPK signaling pathway in QZF group.

To reveal the potential mechanism of QZF on MASH, the RNA-seq analysis of liver tissue was performed. Based on the trend of gene changes in the MASH and QZF treatment groups, we further categorized the gene profiles into eight groups (Figure 3A). Genetic profiles 2 and 5 were the focus of our attention. This is because the results showed that the transcriptional profiles were converted to the expression profiles of normal mice after treatment with the QZF (Figure 3B). Further, the GO and KEGG analysis enriched in metabolic pathways (Figures 3C,D). Gene Set Enrichment Analysis (GSEA) revealed that the gene set associated with positive regulation of autophagy and AMPK signaling pathway were enriched in the experimental group (Figures 3E,F).

### 3.3 QZF alters hepatic metabolites profiles in MASH

**FIGURE 4:**
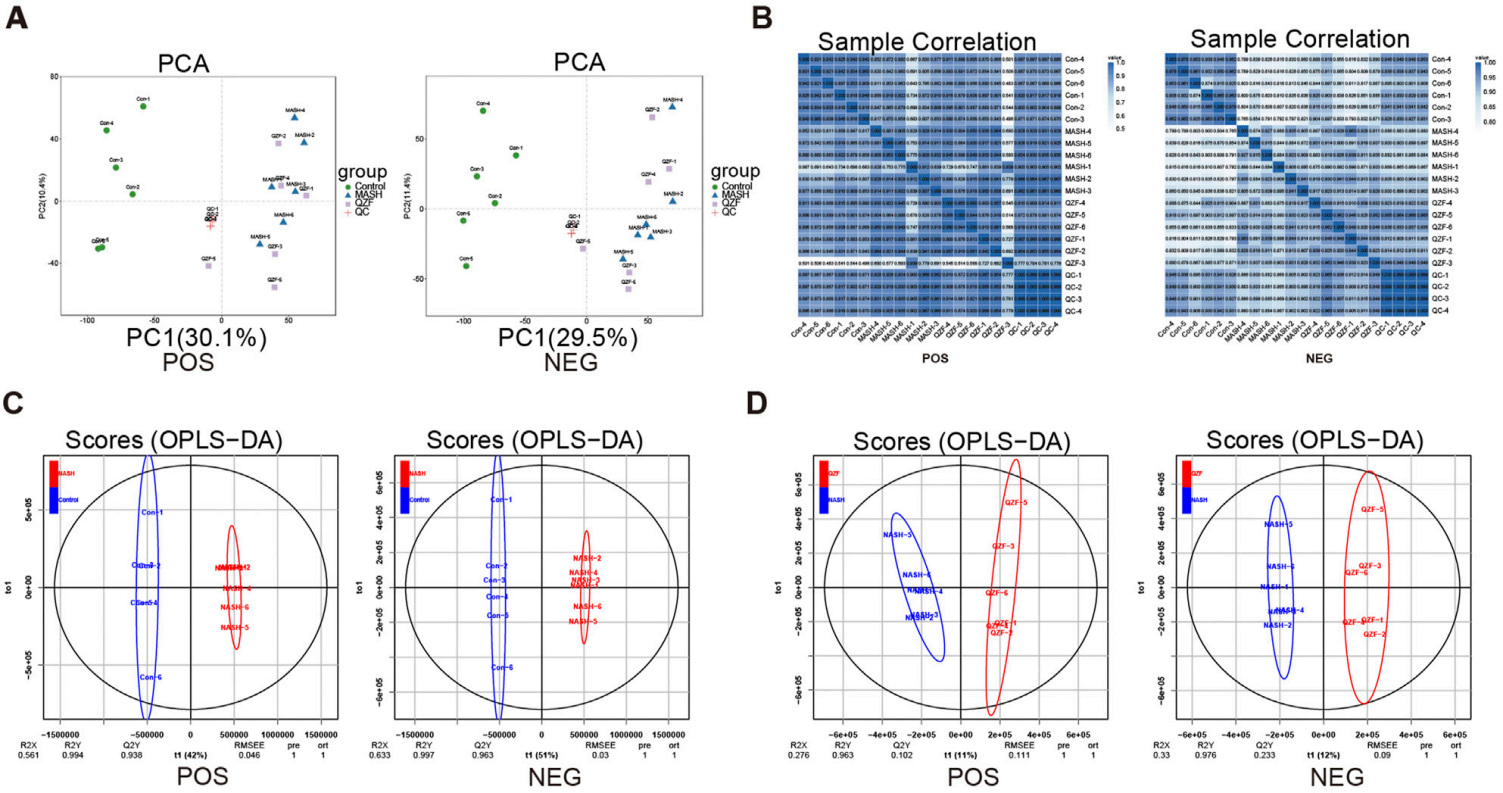
Hepatic metabolomics profiles under QZF treatment. **(A)** PCA score plots (positive/negative ion modes). **(B)** Sample correlation heatmap (Pearson) showing high intra-group consistency and inter-group separation, with QZF exhibiting partial metabolic shift. **(C,D)** OPLS-DA models (positive ion mode) identifying group-specific metabolic alterations. Abbreviation: OPLS-DA, orthogonal partial least squares discriminant analysis.

To investigate global metabolic alterations, untargeted metabolomics analysis was performed in liver tissues in both positive and negative ion modes. In both ion modes, the Control group clustered tightly on the left side of the PCA space, while MASH samples were clearly separated along the first principal metabolite (PC1), indicating significant metabolic disorder. Notably, samples from the QZF-treated group formed a distinct cluster that partially overlapped with the MASH group but shifted toward the Control group, suggesting a partial metabolic restoration upon QZF treatment (Figure 4A). Heatmaps of sample-to-sample Pearson correlation coefficients revealed high intra-group consistency and distinct inter-group separations. Samples within the Control, MASH, and QZF-treated groups exhibited strong internal correlations, while clear dissimilarities were observed between groups. The QZF group showed moderate similarity to MASH and partial separation, suggesting a metabolic shift upon treatment (Figure 4B). To further explore group-specific metabolic alterations, orthogonal partial least squares discriminant analysis (OPLS-DA) was performed under positive ion mode (Figures 4C,D). Clear metabolic separation was observed between the Control and MASH groups in both OPLS-DA score plots. Similarly, OPLS-DA demonstrated clear separation between the MASH and QZF-treated groups, suggesting significant metabolic remodeling after treatment.

**FIGURE 5:**
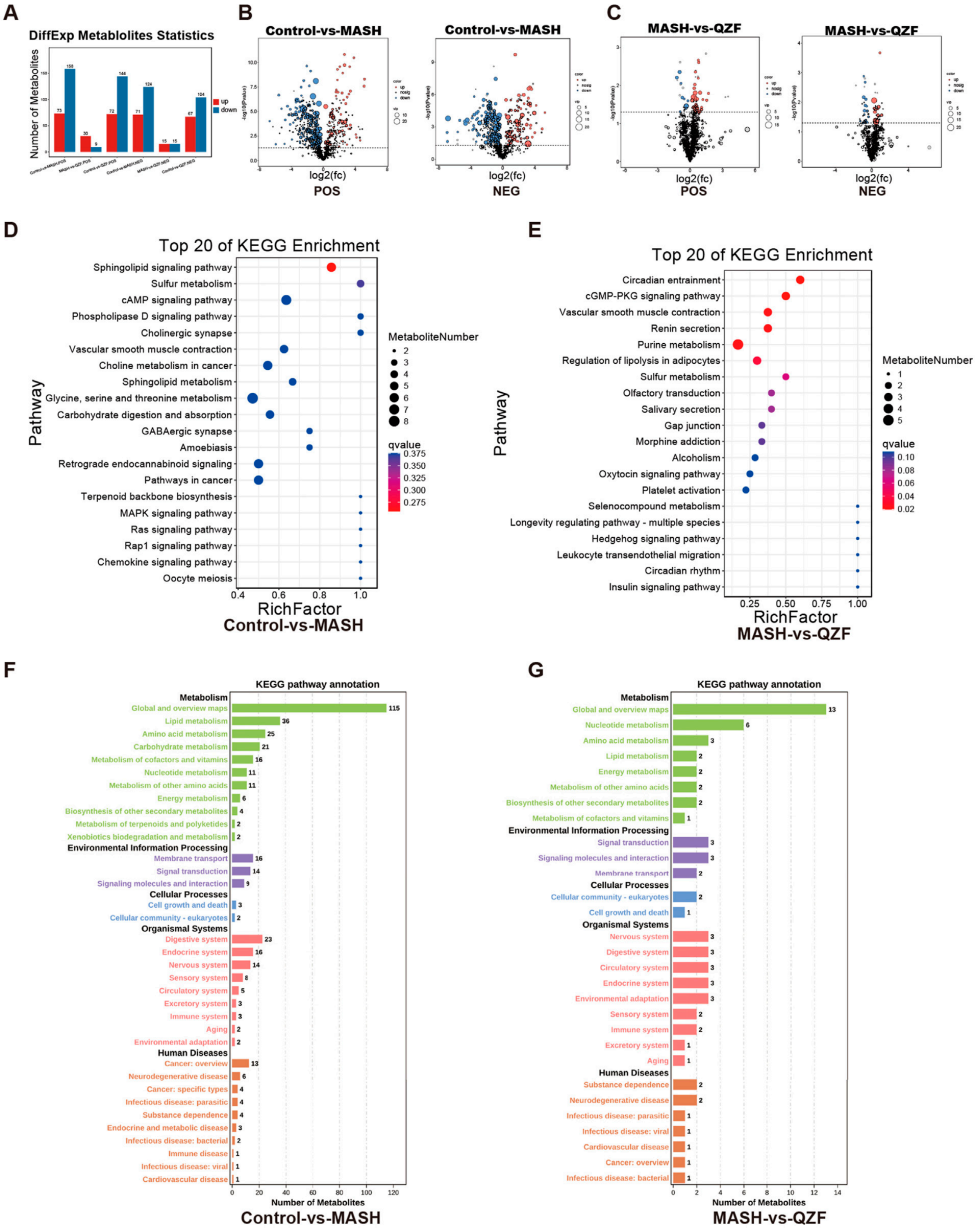
Key metabolic pathways altered by QZF. **(A–C)** Volcano plots demonstrate severe metabolic dysregulation in MASH versus Control with 231 significantly altered metabolites (73 upregulated, 158 downregulated), contrasted by only 39 differential metabolites in MASH versus QZF-treated groups, indicating therapeutic metabolic reset; **(D–G)** Subsequent pathway enrichment analysis (KEGG).

A total of 231 significantly altered metabolites (73 upregulated and 158 downregulated) were identified in the Control vs. MASH comparison under positive mode, reflecting profound metabolic disruptions in the MASH group. In contrast, only 39 differential metabolites were found between the MASH and QZF groups, suggesting partial metabolic normalization following QZF treatment (Figures 5A–C). Pathway enrichment analysis identified a series of differentially enriched metabolites, highlighting the important involvement of metabolic pathways, such as lipid metabolism, amino acid metabolism, and purine metabolism (Figures 5D–G).

### 3.4 LC-MS/MS-metabolomics integration reveals QZF’s bioactive metabolites and Anti-MASH mechanisms

**FIGURE 6:**
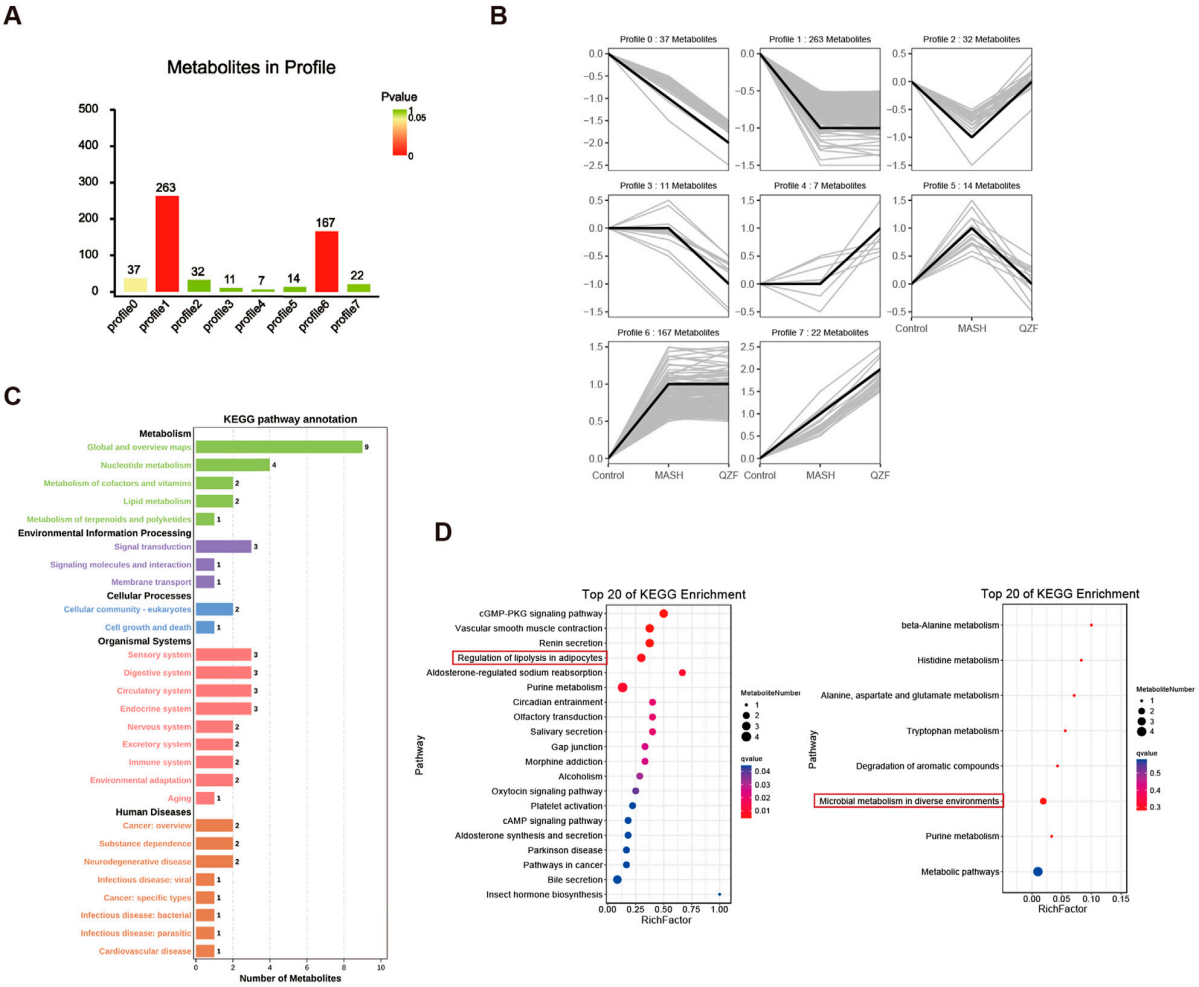
Hepatic metabolome dynamics in MASH and QZF intervention. **(A)** Multivariate trajectory analysis identifying eight metabolic response patterns to MASH and QZF. **(B)** Therapeutic efficacy weighting prioritizes Clusters 2/5, showing full metabolic normalization post-QZF treatment. **(C)** Profile 2: KEGG metabolic pathway dominance. **(D)** Enriched microbial-metabolite crosstalk in Profile 2 post-QZF treatment.

Multivariate trajectory analysis of the hepatic metabolome revealed eight characteristic response patterns to MASH pathogenesis and QZF treatment (Figure 6A). Clusters 2 and 5 were identified as critical regulatory modules through therapeutic efficacy weighting algorithms, demonstrating complete metabolic reprogramming toward physiological steady-state conditions post-intervention (Figure 6B; Supplementary Table S3). Specifically, Profile 2 clustering patterns revealed that its KEGG pathway classification was mainly focused on metabolic pathways, and further analysis showed the enrichment of autophagy-inducing metabolites and mecrobial metabolism after treatment, suggesting activation of protective metabolic programs (Figures 6C,D).

To identify candidate bioactive metabolites responsible for the therapeutic effects of QZF, LC-MS-based metabolic profiling was performed on the QZF extract, there were total 223 active metabolites were identified (Supplementary Table S1).

**FIGURE 7:**
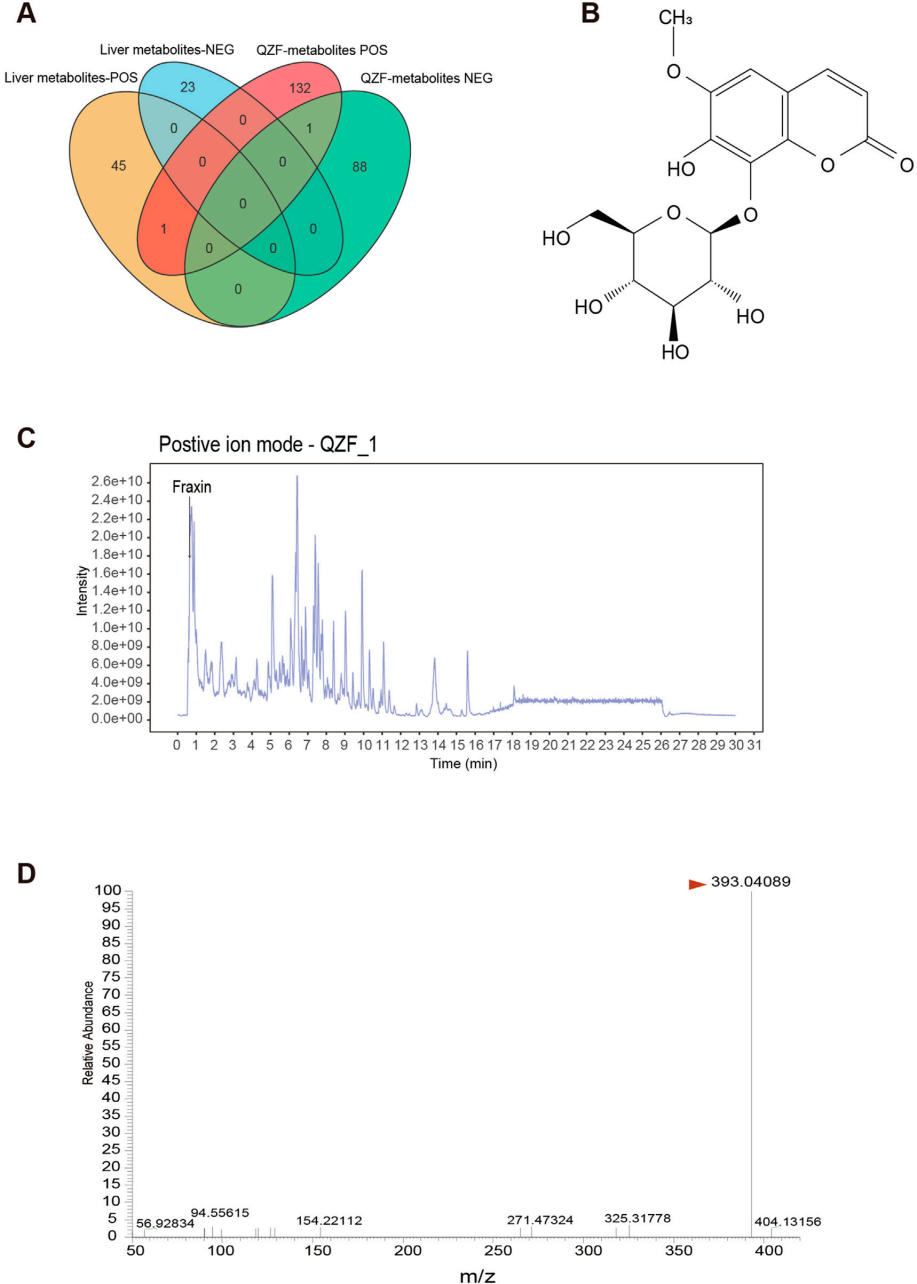
Identification of Fraxin from QZF Extract as a Bioavailable Agent Targeting Hepatic Therapeutic Clusters. **(A)** Venn diagram identifying 2 QZF-derived metabolites overlapping with *in vivo* therapeutic clusters (Profile2/5), suggesting systemic bioavailability. **(B)** Chemical structural formula of Fraxin. **(C)** LC-MS chromatograms confirming consistent detection of Fraxin in QZF extract. **(D)** Mass-to-charge ratio of Fraxin in QZF extract.

A Venn diagram analysis was conducted to compare the metabolites detected in QZF with those found in the biological samples from Profile2 and Profile5. The analysis revealed that one metabolite—Fraxin—overlapped between these groups (Figure 7A). This overlap suggests that this metabolite may be systemically absorbed and functionally active in the biological context.

Fraxin, a glycoside exhibiting antioxidant and anti-inflammatory activities (Figure 7B), was the focus of this investigation. It was consistently detected in both QZF and animal liver. Its retention time matched well with a major peak in the QZF chromatogram, supporting its identity and abundance (Figures 7C,D). These findings suggest that Fraxin may serve as a candidate bioactive metabolite contributing to the observed therapeutic effects of QZF.

### 3.5 Fraxin ameliorates hepatic steatosis via AMPK-autophagy activation

**FIGURE 8:**
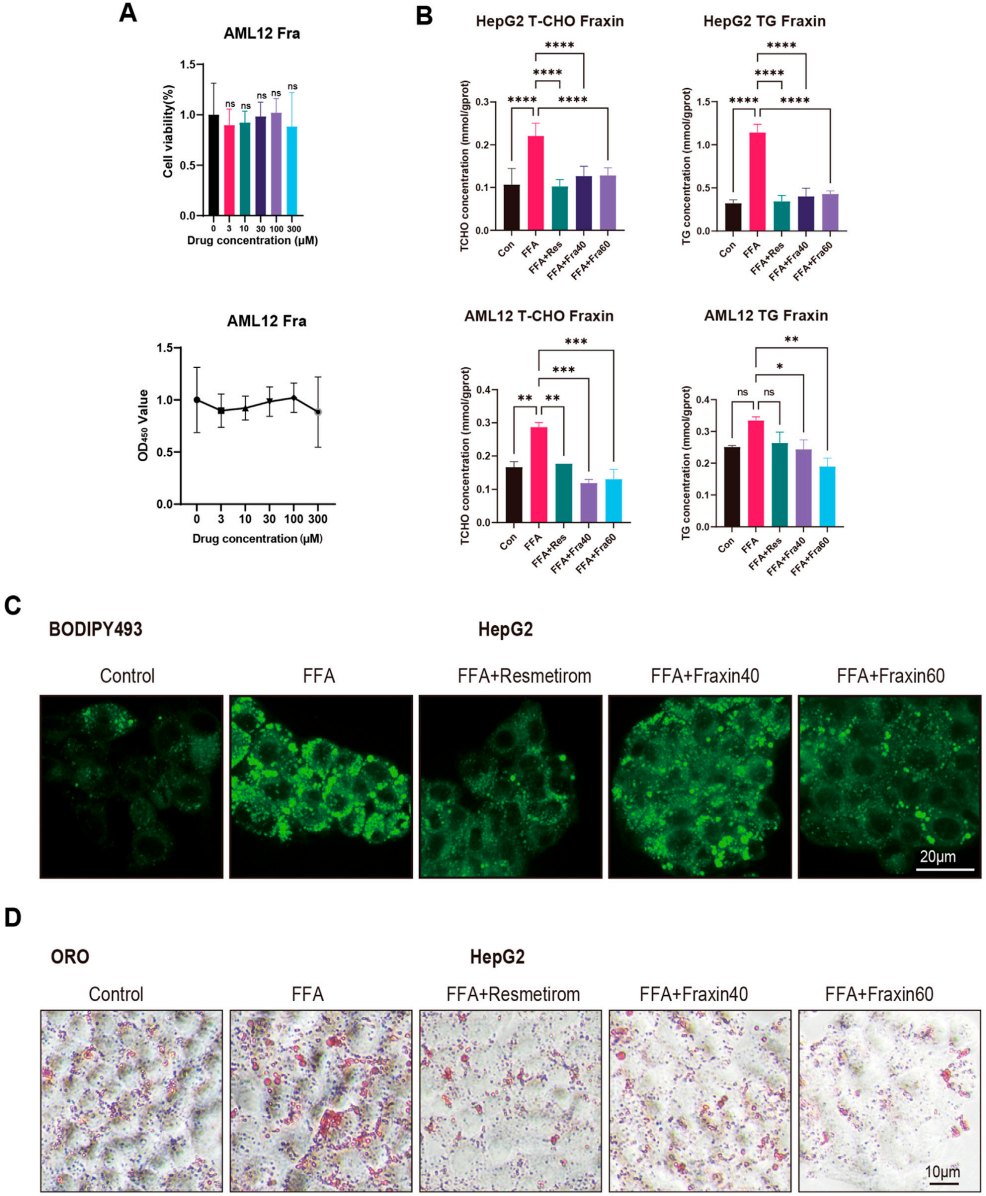
Fraxin ameliorates hepatic steatosis *in vitro*. **(A)** Cell viability assay: No cytotoxicity in AML12 hepatocytes across Fraxin doses (0–120 μM). **(B)** Biochemical quantification: Fraxin dose-dependently decreases TC/TG in FFA-induced HepG2/AML12 steatosis models (P < 0.01 vs. FFA). **(C,D)** BODIPY/Oil Red O staining shows Fraxin (60 μM) abolishes lipid accumulation.

To assess the safety of Fraxin, cell viability assays were conducted on AML12 hepatocytes treated with increasing concentrations of Fraxin. And no significant cytotoxicity was observed across all doses, indicating a favorable safety profile (Figure 8A). Next, the lipid-lowering effect of Fraxin was evaluated using a free fatty acid (FFA)-induced steatosis model in HepG2 cells and AML12 cells. Enzymatic biochemical quantification of cellular lysates revealed that total cholesterol (TC) and triglyceride (TG) levels were significantly decreased following Fraxin treatment (Figure 8B). Moreover, BODIPY staining revealed massive intracellular lipid droplet accumulation in the FFA group, which was substantially reduced by Fraxin in a dose-dependent manner (Figure 8C). Notably, treatment with Fraxin at 60 μM almost completely abolished lipid accumulation, with effects comparable to the positive control Resmetirom. Oil Red O staining showed a marked reduction in lipid droplet accumulation in the treatment group compared to the Control group, indicating effective amelioration of hepatic steatosis (Figure 8D). These findings suggest that Fraxin effectively attenuates hepatic lipid accumulation *in vitro*, supporting its potential as a therapeutic agent for MAFLD.

**FIGURE 9:**
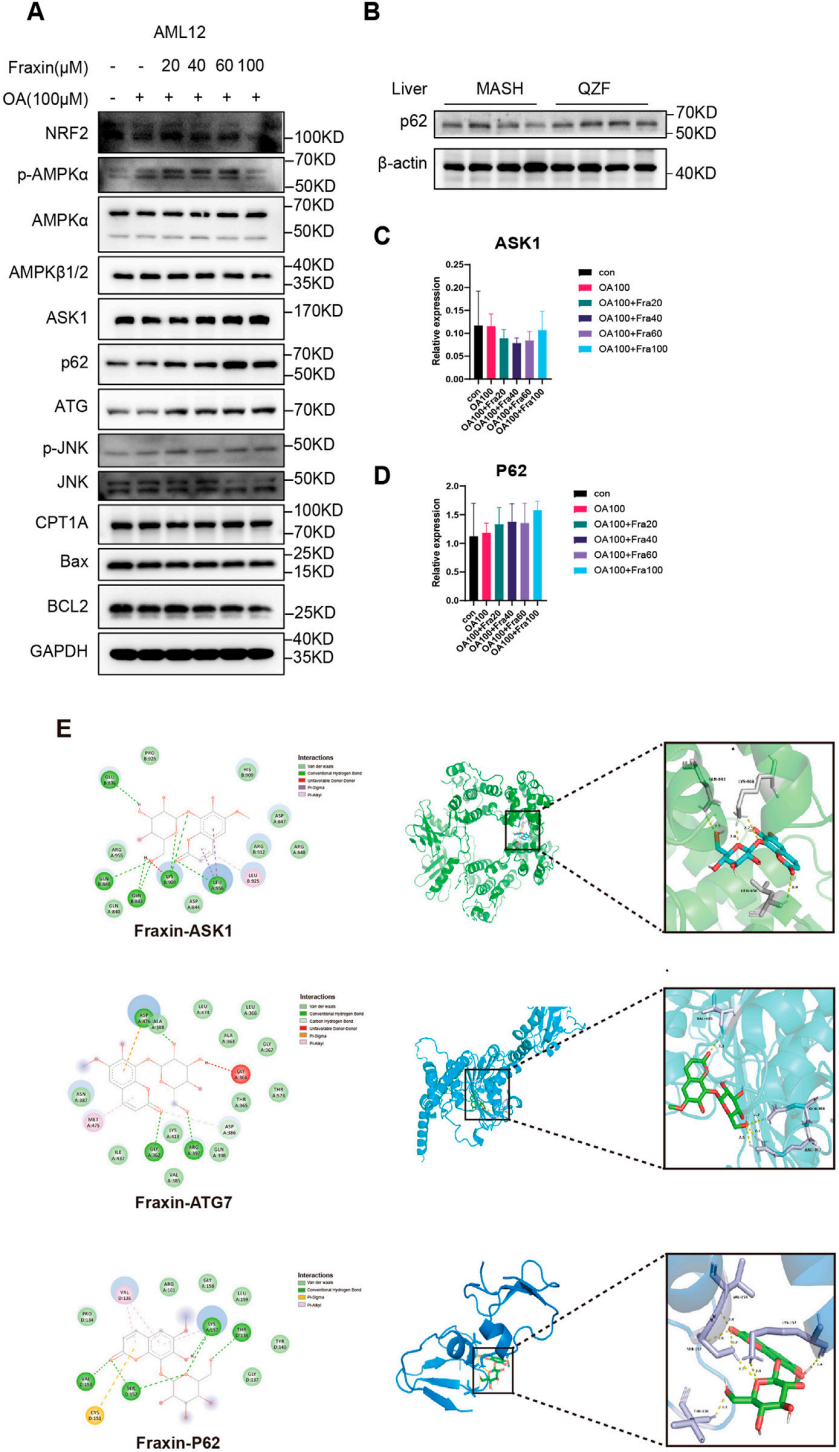
Fraxin ameliorates hepatic steatosis via AMPK-autophagy activation. **(A)** Western blot detection of AMPK-autophagy pathway related protein in Fraxin treated AML12 cell. **(B)** Western blot detection of P62 protein in QZF treated mice liver samples (n = 4). **(C,D)** qPCR detection of AMPK-autophagy pathway related genes in Fraxin treated AML12 cell. **(E)** Molecular docking analyse of Fraxin with AMPK-autophagy pathway related protein.

To elucidate the mechanism by which Fraxin alleviates lipid accumulation, key signaling proteins were analyzed by Western blot in OA-induced AML12 cells. Fraxin dose-dependently increased the expression of NRF2, phosphorylation of AMPKα and total AMPKα, indicating activation of the AMPK pathway. Fraxin treatment also elevated Autophagy-related proteins ATG7, ASK1, and phosphorylated JNK (p-JNK). Additionally, P62 expression increased in both *in vitro* (AML12 cells) and *in vivo* (liver tissue from QZF-treated mice) (Figures 9A,B). and no significant change was observed in expression of pro-apoptotic proteins such as Bax. qPCR assay showed that Fraxin treatment did not significantly affect the transcript levels of ASK1 and P62, suggesting that Fraxin may play a role through post-translational regulation (Figures 9C,D).

Following GSEA of RNA-seq data that suggested QZF-induced upregulation of autophagy pathways, the molecular interactions between Fraxin and a pivotal autophagy-associated protein were investigated *in silico* using molecular docking simulations. Molecular docking revealed significant binding affinities between Fraxin and key autophagy-related proteins ASK1, ATG7, and P62. As depicted in Figure 9E, the most stable complexes, indicated by their binding free energies, were Fraxin-ASK1 (−8.3 kcal/mol), Fraxin-ATG7 (−7.5 kcal/mol), and Fraxin-P62 (−6.3 kcal/mol).

These findings collectively support that Fraxin exerts its hepatoprotective effect by activating AMPK signaling, enhancing autophagy, and suppressing oxidative stress and apoptosis.

## 4 Discussion

Metabolic dysfunction-associated steatohepatitis (MASH) represents a multifactorial global health challenge. Current clinical treatments for MASH demonstrate limited efficacy and frequently cause significant adverse effects (Tang et al., 2025). In this study, we demonstrated that QZF alleviates metabolic dysfunction-associated steatohepatitis (MASH) through coordinated multi-system modulation. The gut–liver axis emerged as a pivotal therapeutic target, evidenced by the QZF-induced recovery of microbial diversity and suppression of pro-steatotic genera such as *Enterococcus*. Gut microbiota dysbiosis is closely associated with the pathogenesis of MASH. Although current studies on gut microbial composition in MASH patients and animal models yield inconsistent results, a consensus exists that at the phylum level, MAFLD patients exhibit reduced Bacteroidetes alongside increased Firmicutes and Proteobacteria (Aron-Wisnewsky et al., 2020). At the family level, *Rikenellaceae* is diminished while Enterobacteriaceae is elevated (Aron-Wisnewsky et al., 2020). At the genus level, *Anaerobacterium*, *Coprococcus*, *Eubacterium*, *Faecalibacterium*, and *Prevotella* are decreased, whereas *Escherichia*, *Dorea*, and *Peptoniphilus* are significantly enriched (Boursier et al., 2016). Elevated abundance of Gram-negative bacteria in the gut—such as *Enterobacter*, *Escherichia*, and *Shigella*—has been implicated in MASH development (Frost et al., 2021). These Gram-negative bacteria release lipopolysaccharides (LPS) through lysis, which disrupts the host’s intestinal epithelial barrier. In obese individuals with fatty liver and high-fat diet-fed mice, gut microbiota analysis reveals increased proportions of *Firmicutes* (e.g., Dorea, *Lactobacillus*, *Lachnospiraceae*, and *Clostridioides*) (Le Roy et al., 2013; Raman et al., 2013). Notably, *Firmicutes* exhibit high metabolic efficiency in energy extraction during dietary degradation (Michail et al., 2015). Additionally, elevated levels of *Erysipelotrichaceae* are observed in the gut microbiota of low-choline diet-fed fatty liver patients and methionine-choline-deficient (MCD) diet-fed mice. *Erysipelotrichaceae* exacerbates hepatic toxicity by converting choline into methylamines, thereby reducing host choline bioavailability and impairing hepatic secretion of very-low-density lipoprotein (VLDL), ultimately contributing to liver injury (Chen et al., 2020). In this study, the relative abundance of *Enterococcus* was markedly elevated in CDAHFD diet-fed mice, which were notably suppressed by QZF. It has been reported that a significant overrepresentation of *Enterococcus* spp. in pediatric cohorts with concurrent obesity and NAFLD (Wei et al., 2024). *Enterococcus faecium B6* (*Enterococcus faecium B6*) could synthesize tyramine, a bioactive metabolite implicated in PPAR-γ activation, thereby driving transcriptional cascades linked to hepatic lipid deposition, inflammatory infiltration, and fibrogenesis (Wei et al., 2024). We also observed that the model group clustered closely with the QZF group but remained distant from the normal group. The proximity between the Model and QZF groups in the PCA plot implies that QZF treatment did not fully restore the gut microbiota structure to that of the Normal group. This suggests that QZF may exert its therapeutic effects not solely by normalizing the microbiota, but also through other mechanisms such as modulating metabolic pathways.

Importantly, untargeted metabolomics revealed the activation of autophagy-associated metabolic pathways, suggesting that QZF reprograms the hepatic metabolic environment toward a protective state. This was further supported by RNA-seq findings, which uncovered upregulation of proteins involved in autophagy, lipid transport, and oxidative stress response. Autophagy, a conserved intracellular degradation pathway for organelles and proteins, encompasses three distinct subtypes: microautophagy, chaperone-mediated autophagy, and macroautophagy (Mizushima and Komatsu, 2011). These processes collectively maintain cellular homeostasis by delivering degradation substrates to lysosomes for proteolytic recycling (Fujiwara et al., 2013). Autophagy exhibits substrate diversity in cellular metabolite degradation: beyond proteolysis to replenish free amino acids, it facilitates the mobilization and hydrolysis of stored lipids and glycogen (Leung et al., 2016; Ezaki et al., 2011). Selective autophagy subtypes—including mitophagy (mitochondrial autophagy), reticulophagy (endoplasmic reticulum autophagy), and pexophagy (peroxisomal autophagy)—precisely regulate the quantity and functionality of these organelles (Singh et al., 2009; Kuma et al., 2004). Furthermore, autophagy mediates the selective degradation of enzymes and proteins involved in glycolysis, lipophagy, and catabolic pathways, thereby modulating the activity of these metabolic cascades (Cecco et al., 2008). Through these multifaceted mechanisms, autophagy engages in hepatic metabolic regulation, underpinning its critical role in diverse liver pathologies, particularly MASH (Hazari et al., 2020). Notably, metabolic syndrome-associated pathological features—including obesity, hyperglycemia, and dyslipidemia—exert deleterious effects on autophagic flux. Hepatic insulin resistance and lipid overload disrupt autophagic-lysosomal function, manifesting as impaired lipid clearance and sustained endoplasmic reticulum stress (ERS) (Liu et al., 2009). Conversely, autophagy deficiency exacerbates insulin resistance, ERS, and lipid metabolic dysregulation, establishing a mutually reinforcing pathological loop between autophagic dysfunction and MAFLD that accelerates MASH progression (Rodriguez-Navarro et al., 2012; Koga et al., 2010). These findings underscore autophagy’s pleiotropic role in MASH pathogenesis and highlight the therapeutic potential of enhancing hepatocyte-specific autophagic activity for MASH amelioration.

Histological analysis confirmed the functional outcomes of these molecular changes, as evidenced by reduced lipid droplet accumulation and significant improvements in serum lipid profiles. Taken together, these results suggest that QZF exerts a multi-faceted effect on MASH pathophysiology, involving microbiome reshaping, metabolic reprogramming, and autophagy induction. Moreover, the integration of multi-omics data highlights the potential of QZF as a systems-level therapy for complex metabolic disorders like MASH.

A key limitation of this study is the relatively high dose of QZF required to achieve therapeutic efficacy, a common challenge in phytopharmacology due to the low concentration of active principles. This is directly attributable to the nature of the crude extract used, where the bioactive metabolite(s) are present in low abundance. It is important to note that while interspecies dose conversion based on body surface area is a valid approach for drugs with low human starting doses (Reagan-Shaw et al., 2008; Xu et al., 2002), its applicability diminishes for high-dose preparations, such as traditional botanical drugs decoctions. The resulting concentrations in animal models can become pharmacologically problematic and prone to artifacts, a concern highlighted in a recent consensus statement (Zhan et al., 2019). Consequently, future research will prioritize the bioactivity-guided fractionation and purification of the active metabolites from QZF. This strategy is essential to achieve potent therapeutic effects at substantially lower, pharmacologically conventional doses, which will not only enhance translational potential but also provide a clearer elucidation of the precise molecular mechanisms of action.

Our findings demonstrate that Fraxin ameliorates hepatic steatosis and exerts hepatoprotective effects through a multi-mechanistic framework involving AMPK signaling activation, autophagy enhancement, and suppression of oxidative stress and apoptosis. These results align with prior evidence showing Fraxin’s capacity to mitigate CCl_4_-induced liver injury via inhibition of MAPK/NF-κB-mediated oxidative stress, lipid peroxidation, and inflammatory cascades (Niu et al., 2017), further solidifying its therapeutic potential for hepatic pathologies. Importantly, as a principal bioactive metabolite of the QZF, Fraxin’s autophagy-modulating properties may underpin QZF’s systemic efficacy in MASH. However, the precise interplay between Fraxin-mediated AMPK activation and downstream autophagic flux regulation warrants further mechanistic dissection, particularly in the context of lipid droplet clearance and inflammasome resolution. Future studies should prioritize dose-dependent efficacy profiling, off-target effect assessments, and clinical validation of Fraxin’s therapeutic index. Additionally, mechanistic investigations into its role within QZF’s phytochemical network could uncover novel combinatorial strategies for treating multifactorial hepatic disorders.

**FIGURE 10:**
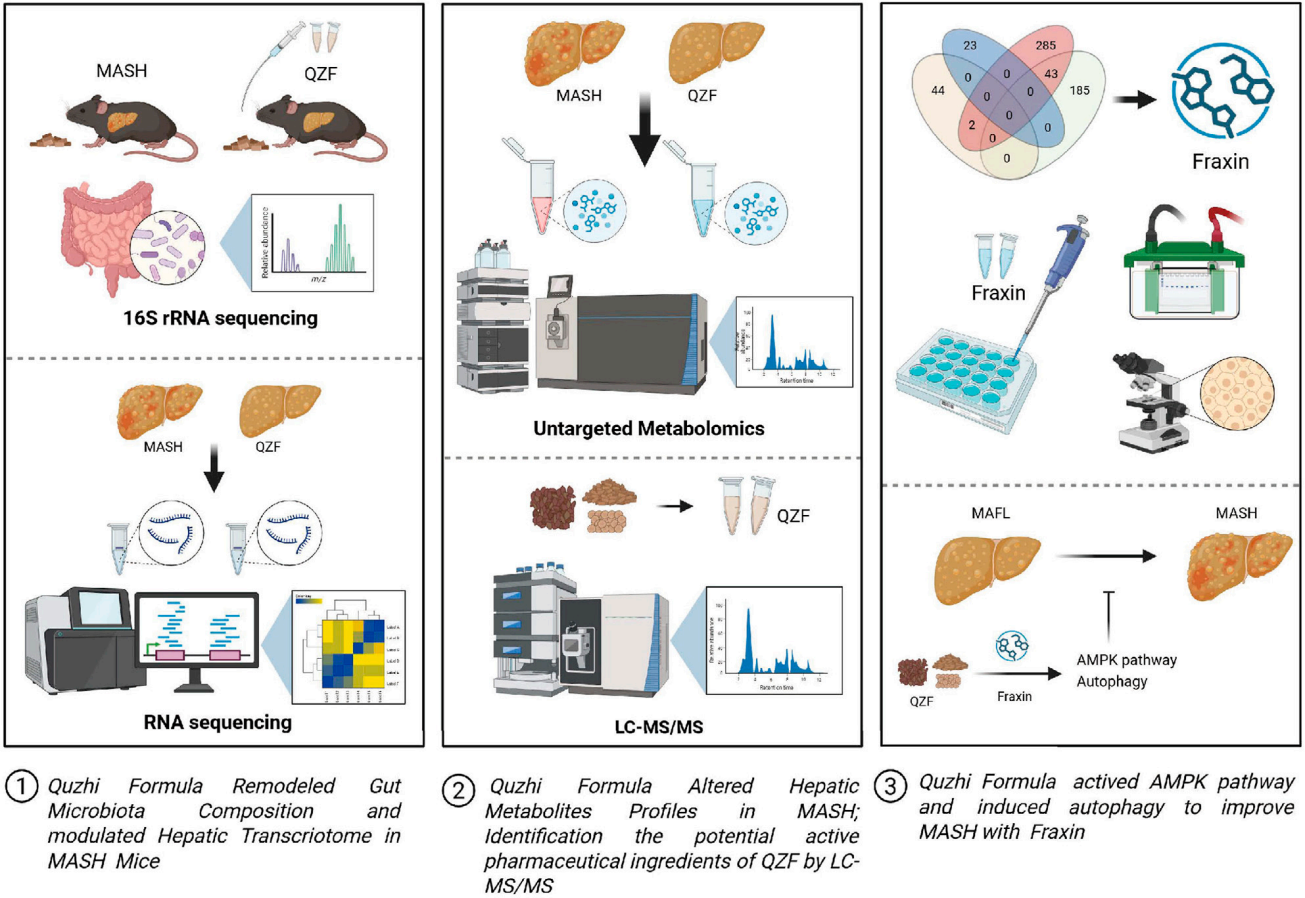
Graphical summary of the study. QZF decoction alleviates MASH by modulating gut microbiota, lipid metabolism, and Fraxin-driven autophagy. This graphical summary was generated by BioRender (https://biorender.com/).

This study demonstrates that QZF ameliorates MASH through multi-system regulation, primarily targeting the gut-liver axis by restoring microbial diversity, suppressing pro-steatotic *Enterococcus*, and activating AMPK/Autophagy-associated pathways to reprogram hepatic metabolism through active metabolite Fraxin. Integrated multi-omics and histological analyses confirm QZF’s efficacy in reducing lipid accumulation, improving serum lipid profiles, and inducing protective molecular mechanisms, positioning it as a systems-level therapeutic candidate for complex metabolic disorders (Figure 10).

## Data Availability

The data presented in the study are deposited in the National Genomics Data Center (NGDC) repository, accession number CRA026321. Any other data are available from the corresponding author on reasonable request.

## Supplementary material

SUPPLEMENTARY FIGURE S1 The LC–MS fingerprint of the QZF extract. **(A)** The LC–MS fingerprint of QZF extract in both ion mode showed good instrument precision, with RSDs of relative retention time and peak area at 0.00%–0.22% and 1.06%–1.43%, respectively. The cosine similarity algorithm was used to assess similarity between samples and common peaks, both overall and within sample groups. **(B)** Metabolites identified in QZF extract were marked by number.

SUPPLEMENTARY FIGURE S2 The low dosage of QZF (4.1666 g/kg) did not alleviate hepatic steatosis in a MASH mouse model. Male C57BL/6 SPF mice (6 weeks old) were fed a CDAHFD for 3 weeks to induce MASH, followed by daily oral gavage of either the low-dose QZF (4.1666 g/kg) or a PBS vehicle for an additional 4 weeks. Mice were euthanized at the end of week 7 for liver collection. Representative liver sections stained with **(A)** H&E and **(B)** Oil Red O are shown. Graphs depict the NAFLD Activity Score (NAS) and the semi-quantitative analysis of lipid deposition from Oil Red O staining, performed using ImageJ. **p < 0.01, ***p < 0.001, ****p < 0.0001 vs. model group (one-way ANOVA). Abbreviations: MASH, metabolic dysfunction-associated steatohepatitis; CDAHFD, choline-deficient, L-amino acid-defined, high-fat diet; H&E, hematoxylin and eosin.

SUPPLEMENTARY FIGURE S3 Acute toxicity assessment of the high-dose QZF in mice. A separate acute toxicity study was conducted to evaluate the safety of QZF. Male C57BL/6 SPF mice (6 weeks old) received a daily oral gavage of QZF at 8.3331 g/kg or PBS vehicle for 4 weeks. **(A)** Representative gross images of the liver. **(B)** Serum levels of alanine aminotransferase (ALT) and aspartate aminotransferase (AST). **(C–F)** Representative histopathological images of H&E-stained sections from the liver **(C)**, kidney **(D)**, heart **(E)**, and lung **(F)**. Data are presented as mean ± SD (n = 9). **p < 0.01, ***p < 0.001, ****p < 0.0001 vs. PBS control group (unpaired two-tailed Student’s t-test).

SUPPLEMENTARY FIGURE S4 The α-diversity analysis. Fecal samples were collected immediately after sacrifice from mice treated with the QZF and from normal controls. Subsequently, 16S rRNA sequencing was performed on these samples. The resulting alpha diversity indices—Shannon **(A)**, Simpson **(B)**, Chao **(C)**, and ACE **(D)**—for both groups and individual samples are presented in this figure.

SUPPLEMENTARY TABLE S1 Active metabolites of QZF decoction.

SUPPLEMENTARY TABLE S2 ConPhyMP-checklist.

SUPPLEMENTARY TABLE S3 Liver metabolites after QZF treated.

## Abbreviations

MASH, metabolic dysfunction-associated steatohepatitis; QZF, Quzhi formula; MAFLD, metabolic dysfunction-associated fatty liver disease; NAFLD, non-alcoholic fatty liver disease; TCM, traditional chinese medicinal; CDAHFD, choline-deficient, l-amino acid–defined, high-fat diet; OA, Oleic acid; PA, Palmitic acid; GSEA, Gene Set Enrichment Analysis; OPLS-D, Orthogonal partial least squares discriminant analysis; FFA, free fatty acid; TC, total cholesterol; TG, triglyceride; LPS, lipopolysaccharides; MCD, methionine-choline-deficient; VLDL, very-low-density lipoprotein; ERS, endoplasmic reticulum stress.
