# Supplementary figures and images for: Multi-omics analysis elucidates the therapeutic mechanisms of the Quzhi formula in metabolic dysfunction-associated steatohepatitis targeting gut microbiota, lipid metabolism, and the role of its metabolite fraxin
Source: Front Pharmacol. 2025 Nov 3;16:1694242. doi: 10.3389/fphar.2025.1694242 (PMC12620365; doi:10.3389/fphar.2025.1694242)

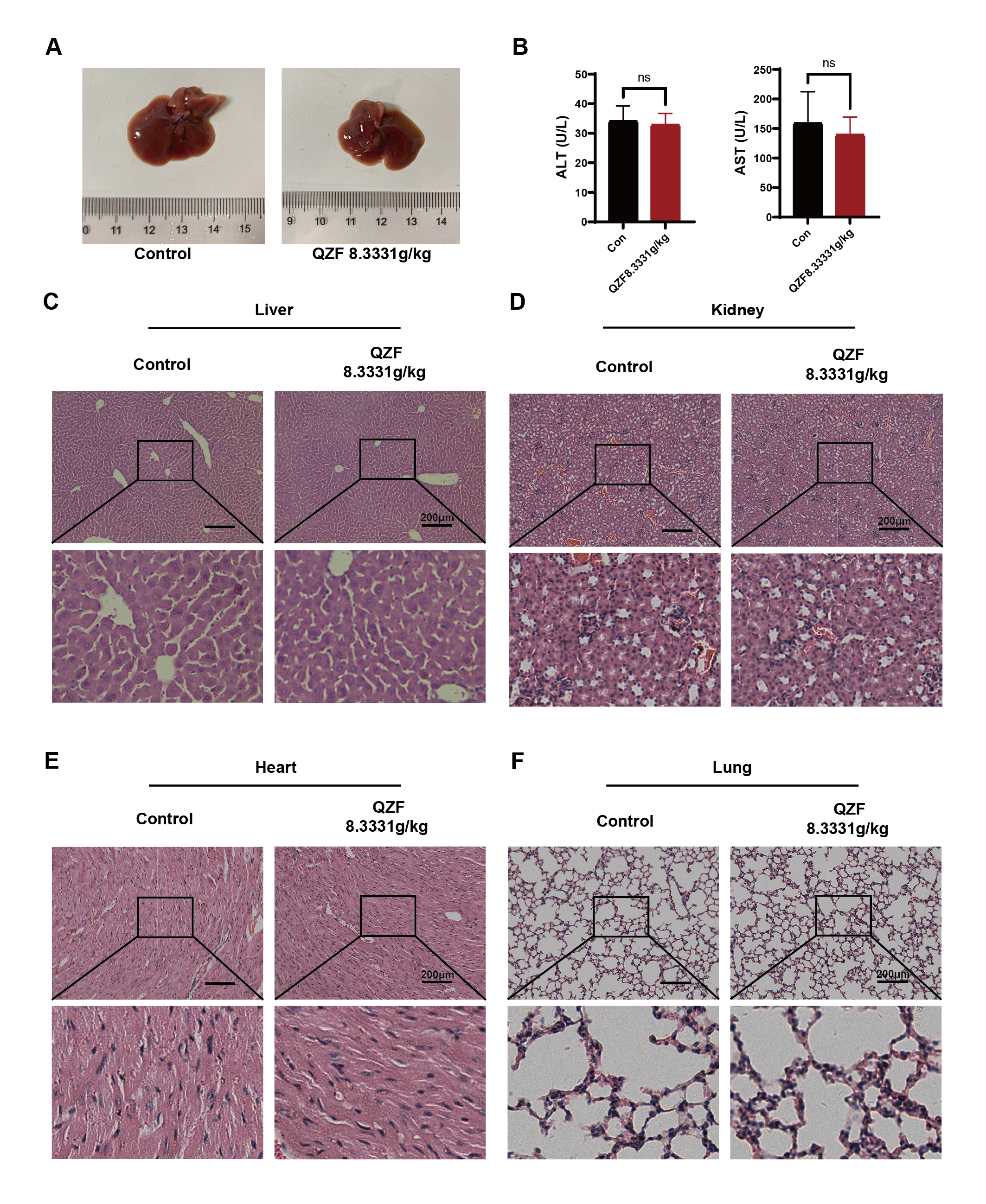

Supplement: Supplementary file 1 [file Image3.jpeg]

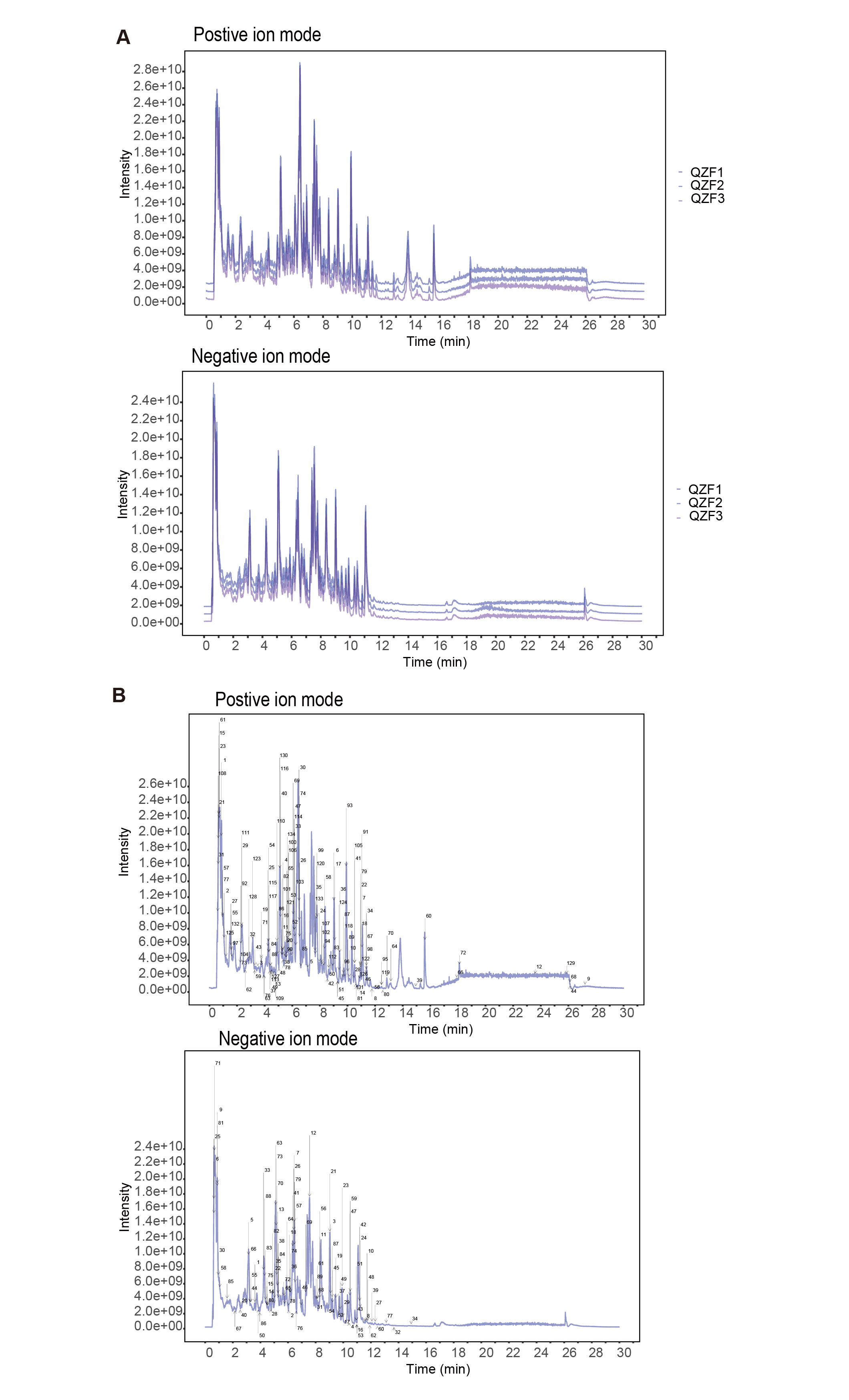

Supplement: Supplementary file 4 [file Image1.jpeg]

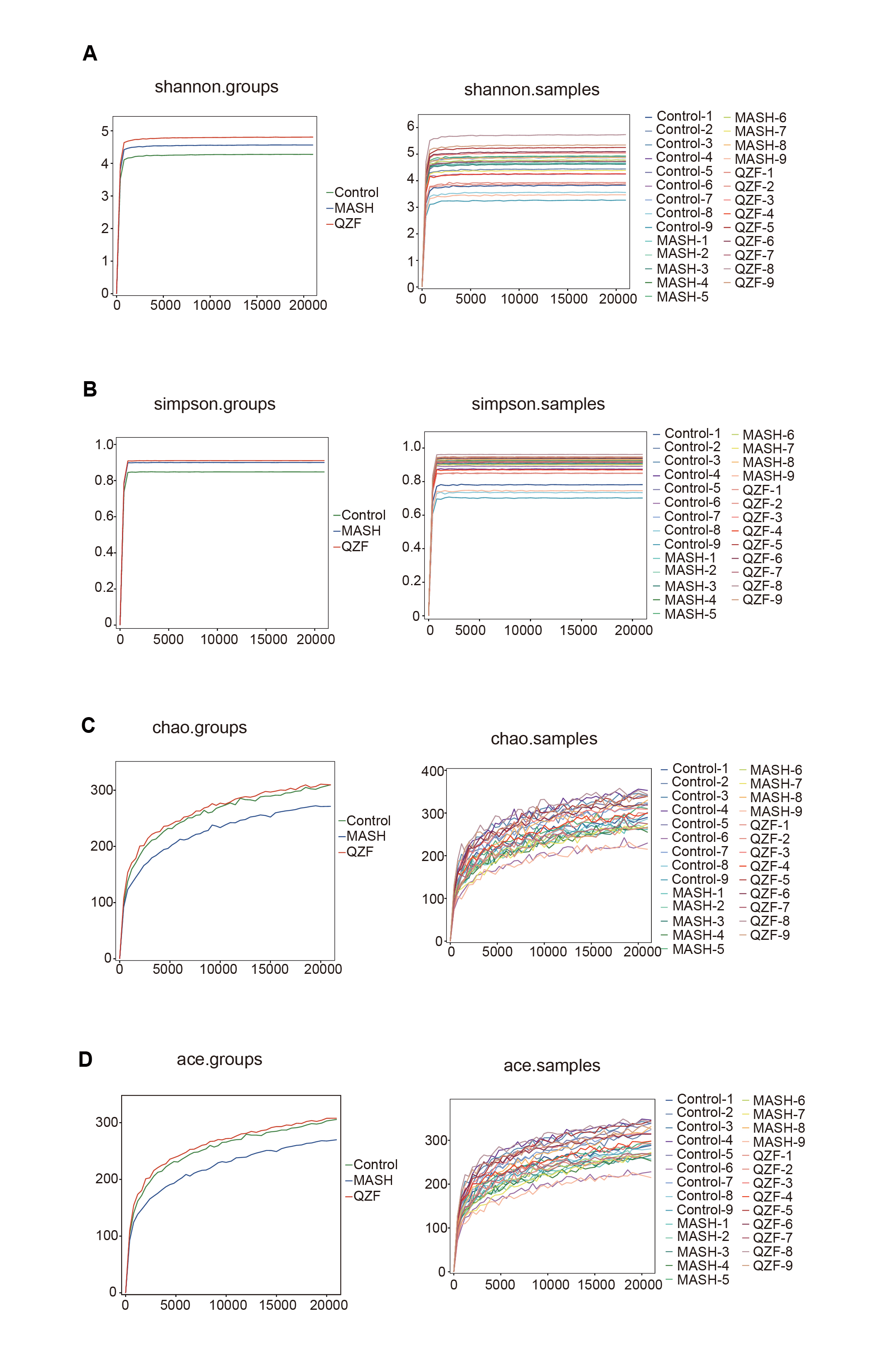

Supplement: Supplementary file 5 [file Image4.jpeg]

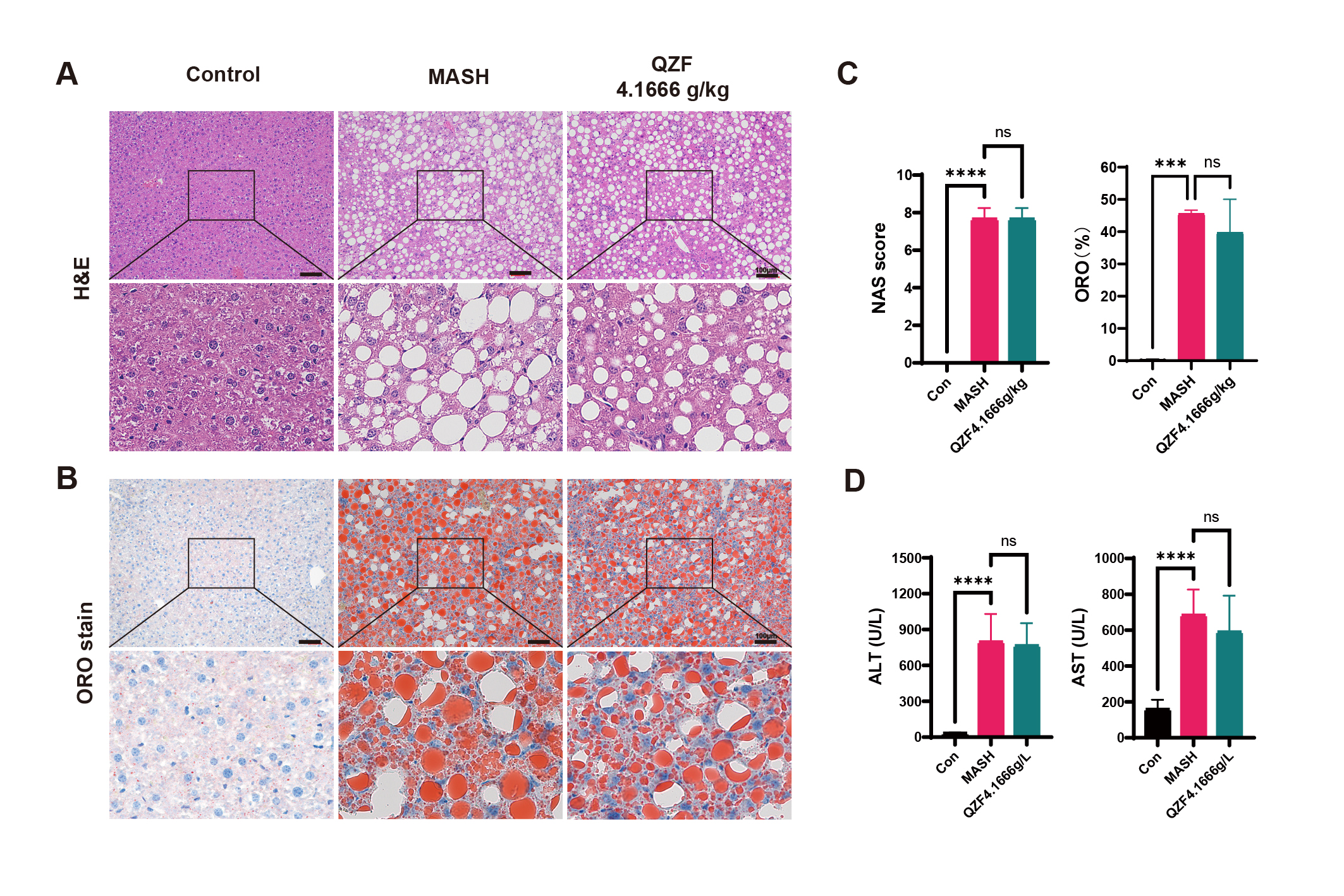

Supplement: Supplementary file 6 [file Image2.jpeg]
